# Variation in time and magnitude of immune response and viremia in experimental challenges with Porcine circovirus 2b

**DOI:** 10.1186/s12917-014-0286-4

**Published:** 2014-12-04

**Authors:** Taylor B Engle, Erin E Jobman, Timothy W Moural, Autumn M McKnite, Justin W Bundy, Sarah Y Barnes, Emily H Davis, Judith A Galeota, Thomas E Burkey, Graham S Plastow, Stephen D Kachman, Daniel C Ciobanu

**Affiliations:** Animal Science Department, University of Nebraska, Lincoln, NE 68583-0908 USA; School of Veterinary Medicine and Biological Sciences, University of Nebraska, Lincoln, USA; Bluegrass Community & Technical College, Lexington, USA; Department of Agricultural, Food and Nutritional Science, University of Alberta, Edmonton, Canada; Department of Statistics, University of Nebraska, Lincoln, USA

**Keywords:** Disease susceptibility, PCV2b, Swine

## Abstract

**Background:**

Porcine circovirus 2 is the primary agent responsible for inducing a group of associated diseases known as Porcine Circovirus Associated Diseases (PCVAD), which can have detrimental effects on production efficiency as well as causing significant mortality. The objective of this study was to evaluate variation in viral replication, immune response and growth across pigs (n = 974) from different crossbred lines. The approach used in this study was experimental infection with a PCV2b strain of pigs at an average of 43 days of age.

**Results:**

The sequence of the PCV2b isolate used in the challenge was similar with a cluster of PCV2b isolates known to induce PCVAD and increased mortality rates. The swine leukocyte antigen class II (*SLAII*) profile of the population was diverse, with nine *DQB1* haplotypes being present. Individual viremia and antibody profiles during challenge demonstrate variation in magnitude and time of viral surge and immune response. The correlations between PCV2 specific antibodies and average daily gain (ADG) were relatively low and varied between - 0.14 to 0.08 for IgM and −0.02 and 0.11 for IgG. In contrast, PCV2 viremia was an important driver of ADG decline following infection; a moderate negative correlation was observed between viral load and overall ADG (r = − 0.35, *P* < 0.001). The pigs with the lowest 10% level of viral load maintained a steady increase in weekly ADG (P < 0.0001) compared to the pigs that had the 10% greatest viral load (P < 0.55). In addition, the highly viremic group expressed higher IgM and IgG starting with d 14 and d 21 respectively, and higher tumor necrosis factor – alpha (TNF-α) at d 21 (P < 0.005), compared to low viremic group.

**Conclusions:**

Molecular sources of the observed differences in viremia and immune response could provide a better understanding of the host factors that influence the development of PCVAD and lead to improved knowledge of swine immunity.

**Electronic supplementary material:**

The online version of this article (doi:10.1186/s12917-014-0286-4) contains supplementary material, which is available to authorized users.

## Background

PCV2 vaccination was proven successful in controlling PCVAD. However, in a standard commercial operation, while the majority of pigs are infected with PCV2b, only a fraction will display PCVAD symptoms [1,2]. Currently, no diagnostic tool is available to identify pigs that have potential susceptibility to PCVAD. As a result, the entire population must be vaccinated in order to protect a fraction of the pigs leading to an increase in production cost. In addition, a research by Cino-Ozuna et al. [3] discovered that acute pulmonary edema, a novel PCVAD syndrome, was associated only with pigs vaccinated for PCV2.

Several studies observed differences in PCVAD susceptibility in several breeds of pigs, with Landrace pigs reported to have increased vulnerability to PCV2 infections compared to Large White, Yorkshire, Duroc and Pietrain pigs [4-6]. Recent research has also shown that host genotype influences PCV2 susceptibility, specifically PCV2 viremia and immune response in experimental infections with PCV2b [7].

Availability of molecular diagnostic approaches that will allow identification of susceptible animals could add another layer of protection in swine operations that experience high pathogen exposure. As part of a research program focused on the influence of swine host genetics to two main viral pathogens (www.swineimprovement.com), at University of Nebraska we initiated a study to uncover phenotypic and genetic predictors of PCVAD susceptibility. Using an expansive array of swine germplasm analyzed, covering a significant proportion of the North-American maternal genetic crossbreds, in this study we evaluated phenotypic profiles, intra-populational variations and relationships between important indicators of PCV2 susceptibility. This represents an essential preliminary step of the future research aimed at uncovering genetic variants that influence the host’s ability to stimulate immune response and reduce disease susceptibility.

## Results and discussions

### The resource population displayed substantial diversity at SLAII locus

The swine leukocyte antigen class II (*SLAII*) is known to be involved in antigen presentation and modulation of immune system [8]. This locus is characterized by extended haplotypes and extremely polymorphic with differences from population to population. The potential role of *SLAII* region in immune response against swine viral pathogens was recently demonstrated by Quantitative Trait Loci (QTL) mapped to the *SLAII* region and associated with PCV2 viremia [7] and with specific antibody response to Porcine reproductive and respiratory syndrome virus (PRRSV) [9]. As a result, we hypothesized that genetic diversity at this locus could result in variation in immune response to PCV2 challenges. The *SLAII* haplotypes were determined by sequencing the coding region of the *DQB1* gene, a member of *SLAII* gene complex, in a sample of pigs (2n = 54) representing all batches. Due to the various genetics used in this study (Additional file 1: Table S1) the genetic profile at this locus was more diverse than in other populations [10] (Table 1). The population included all nine *DQB1* class haplotypes (*01XX* to *09XX*) with the specific *0701* haplotype being predominant (27.8%). In comparison, four to eight haplotypes were identified in a different study across four outbred populations, most having major haplotypes with frequencies ranging from 43.9 to 54.2% [10].

**Table 1:** **Proportion (%) of the** ***DQB1*** **specific Swine leukocyte antigen (** ***SLAII*** **) haplotypes in UNL PCV2-challenged resource population** ^**1**^ **and other populations of outbred pigs** ^**2**^

**Haplotypes**	**UNL (2n = 54)**	**PCV** ^**2**^ **(2n = 104)**	**Big Pig** ^**2**^ **(2n = 186)**	**KSU** ^**2**^ **(2n = 98)**	**MY** ^**2**^ **(2n = 24)**
*01XX*	5.6	9.6	17.2	23.5	4.2
*02XX*	13.0	45.2	30.1	43.9	25.0
*03XX*	11.1	6.7	3.2	6.1	16.7
*04XX*	5.6	6.7	31.2		
*05XX*	11.1	7.7		3.1	
*06XX*	13.0		14.0		
*07XX*	27.8	14.4	2.7	18.4	
*08XX*	3.7	3.8		5.1	54.2
*09XX*	9.3	5.8	1.6		

^1^Determined by sequencing the coding region of the *DQB1* gene in pigs representing all batches.

^2^Data from Ho et al. (2010); PCV, porcine circovirus; KSU, Kansas State University; MY, Meishan x Yorkshire cross.

### The genome of the PCV2b isolate is similar with a cluster of PCV2b strains known to induce PMWS

Variation in strain virulence was demonstrated in natural and experimental challenges with PCV2 [5,11,12]. The PCV2b strain (UNL2014001) used in experimental inoculation was collected from a pig that displayed clinical symptoms of Post-weaning Multisystemic Wasting Syndrome (PMWS), a PCVAD syndrome. The viral DNA genome was sequenced (accession number KP016747) and compared with the genome sequences of a collection of PCV2 isolates reported by Gagnon et al. [13] using CLUSTALW2 alignment software. The PCV2b viral sequence had the highest similarity with a cluster of Quebec PCV2b isolates (e.g., FMV-05-6507, FMV-05-6302, FMV-05-7389) [7] known to induce PMWS and increased mortality rates. The strain that displayed the highest genetic similarity with the UNL strain was FMV-05-6507 strain; a single synonymous nucleotide difference located in the last position of the 42 codon of the capsid gene represents the only variation (G to A) between the genome of the UNL strain and FMV-05-6507 isolates. However, in addition to PCV2, the PMWS cases associated with the cluster of strains isolated from Quebec were found positive for other swine pathogens such as PRRSV (30.8%), Swine Influenza Virus (15.4%), Porcine parvovirus (38.5%), Swine hepatitis E virus (38.5%) and Swine torque teno virus (69.2%) [13]. As a result, coinfection could have been an important determinant of these severe PMWS cases. It is known that PCV2 infection of cells involved in the innate immune response affect host recognition of viral and bacterial antigens leading to susceptibility to secondary infections [14,15]. It is possible that this cluster of strains is very efficient in inhibiting host immune response increasing susceptibility to coinfection. During experimental infection in the current study the mortality was 2.98%, with causes other than PCV2b alone being the most likely sources of death.

Due to inadequate biosecurity and the fact that inactivation of PCV2 with common detergents and disinfectants proved very difficult [16], multiple PCV2 isolates could be identified simultaneously in the same herd [2]. In order to validate the genetics of PCV2b strain as a consistent infectious isolate across batches, viral genomic DNA from random, high and low viral load pigs (n = 18) representing most of the batches were completely sequenced. The sequences of this set of samples were identical with the sequence of PCV2b isolate used for experimental infection.

### The passive IgG was higher in the piglets generated by dams with five or more parities

**Figure 1:**
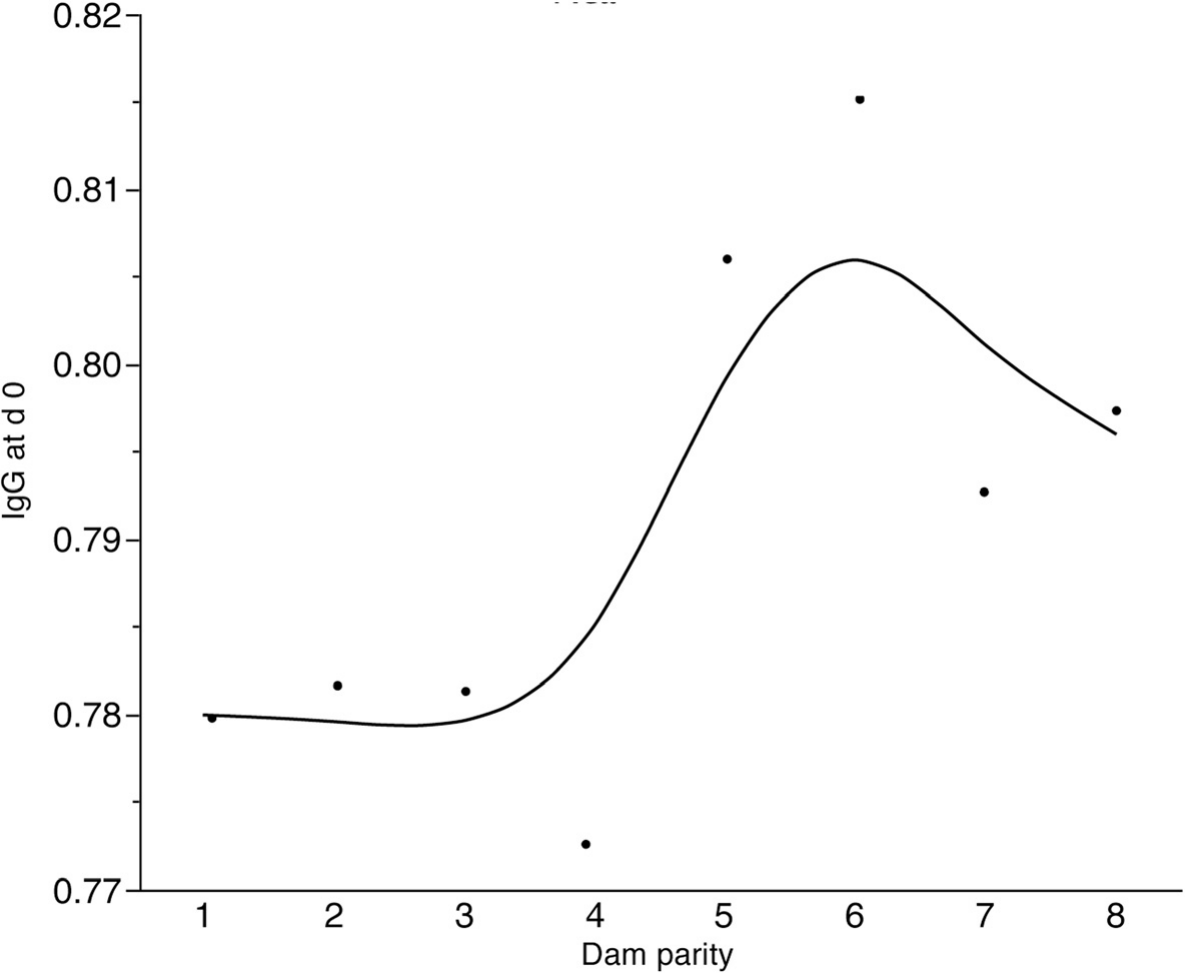
**The influence of dam parity (X) on the level of passive IgG (Y) in experimental piglets.** Concentration of passive IgG tended to increase in piglets generated by older dams (> 4 parities, P > 0.27).

All pigs receiving colostrum acquire antibodies that provide early immunity against PCV2 and other pathogens. The effectiveness is dependent upon the concentration and rate of antibody decline, which in the case of PCV2 typically occurs between 5 to 18 weeks (wk) of age [6]. Previous studies have demonstrated an association between titers of passive (maternal) IgG antibodies and the extent of protection against PCV2 [17]. For instance, low IgG titres were associated with an increased number of PCV2 positive pigs while high IgG titres were associated with lower numbers of PCV2 positive pigs. Understanding the influence of PCV2 specific antibodies in dams and piglets could be useful to prevent disease progression. Analysis of passive antibodies from all candidate piglets prior to infection showed variation in IgG levels. While not significant (*P* > 0.27) the level of passive IgG tended to increase as the dam’s number of parities increases, this was particularly evident after parity four (Figure 1). As a result, PCV2 vaccination of pigs derived from younger dams could be recommended early in life due to lower level of passive IgG.

### Growth rate during experimental infection with PCV2b

**Figure 2:**
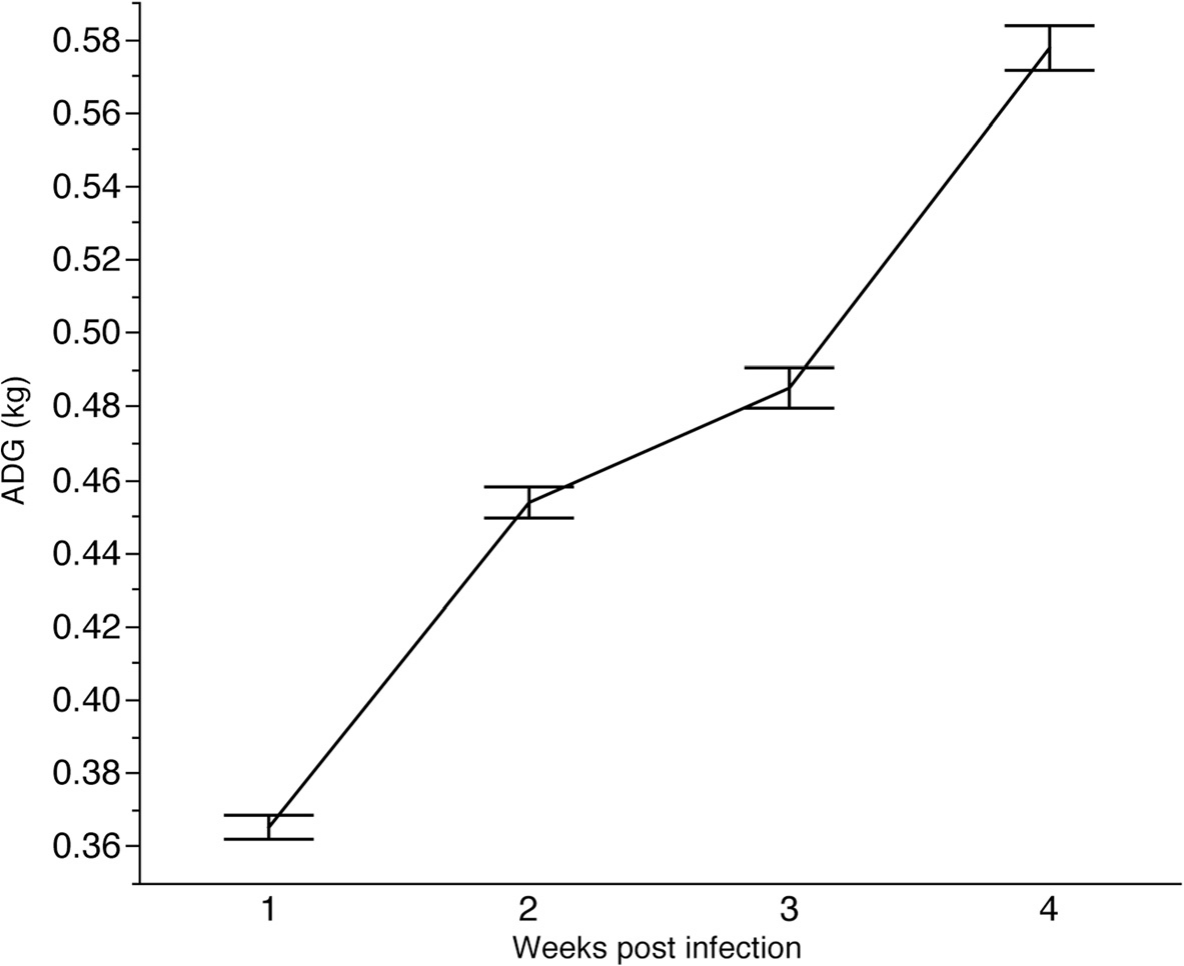
**Variation across time points in ADG (kg) following experimental challenge with PCV2b.**

**Figure 3:**
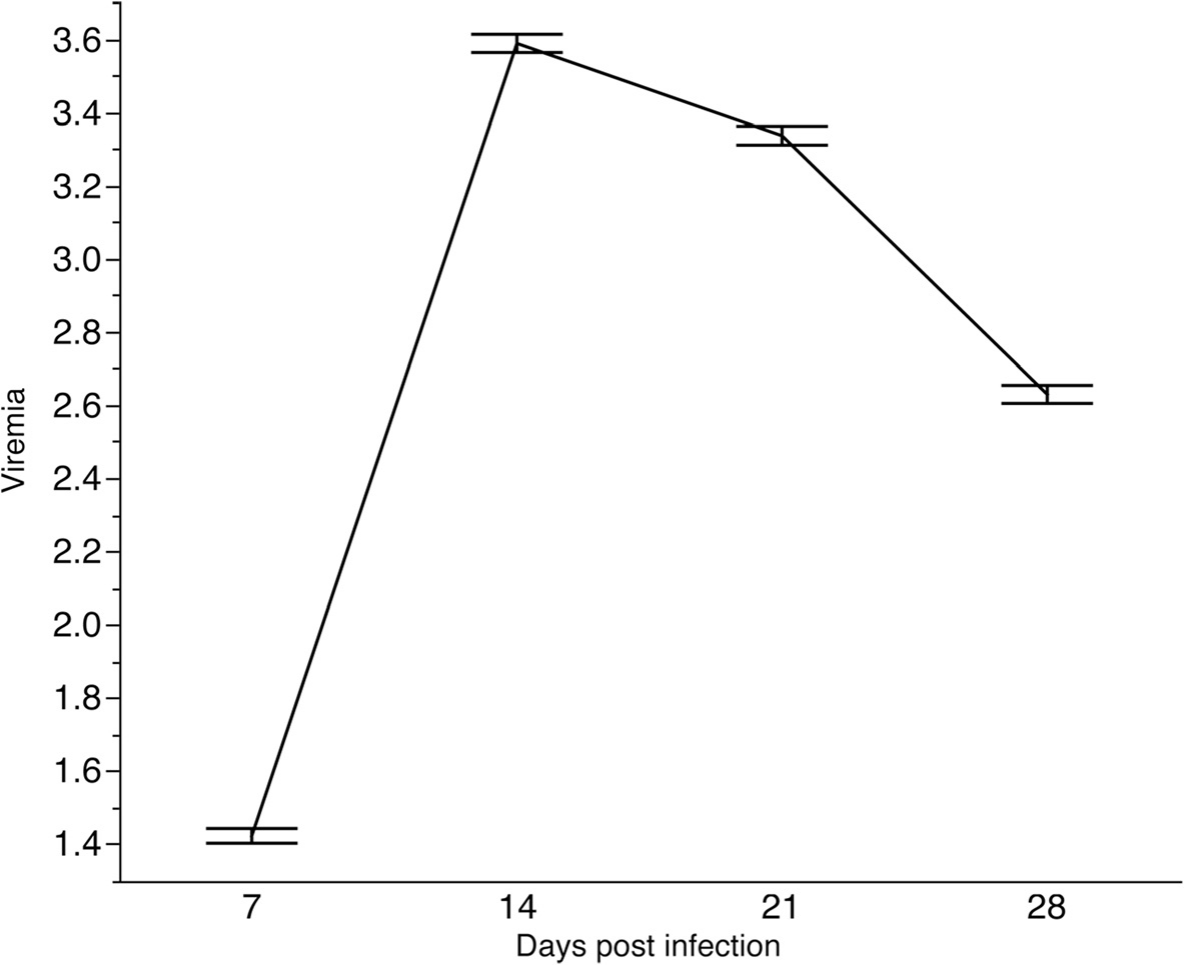
**Variation across time points in PCV2b viremia** * **following experimental challenge with PCV2b.** *measured as number of PCV2 genome copy number/ml of serum (log10).

The wk 3 and wk 4 were the weeks associated with the largest variation in ADG following PCV2b challenge (Table 2). In the last week of challenge the pigs exhibited the largest average ADG, as the immune system started to clear the virus (Figure 2). Previous studies showed that PCV2 vaccinated pigs exhibit consistent increased in ADG during this development period [18]. In this study, average ADG across time points indicates a deviation from this steady increased in weekly ADG due to a plateau in growth observed during wk 3 (Figure 2). This plateau could be a result of viral replication since it coincides with the days characterized by the largest viremia levels (Figure 3).

**Table 2:** **Means and standard deviations (SD) of the indicator traits of PCVAD susceptibility following an experimental infection with PCV2b**

**Trait**	**Mean**	**SD**
ADG wk1 (kg)	0.37	0.10
ADG wk2 (kg)	0.45	0.13
ADG wk3 (kg)	0.48	0.17
ADG wk4 (kg)	0.58	0.18
ADG 0–28 d	0.47	0.10
Viremia d 7*	1.42	0.62
Viremia d 14*	3.59	0.76
Viremia d 21*	3.34	0.78
Viremia d 28*	2.63	0.75
IgM d 7	0.59	0.07
IgM d 14	1.18	0.32
IgM d 21	1.45	0.46
IgM d 28	1.16	0.40
IgG d 7	0.73	0.10
IgG d 14	0.82	0.19
IgG d 21	1.70	0.62
IgG d 28	2.24	0.73
Viral load	68.86	12.35

*measured as number of PCV2 genome copy number/ml of serum (log10).

### Viremia is the main driver of growth decline during PCV2b challenge

**Figure 4:**
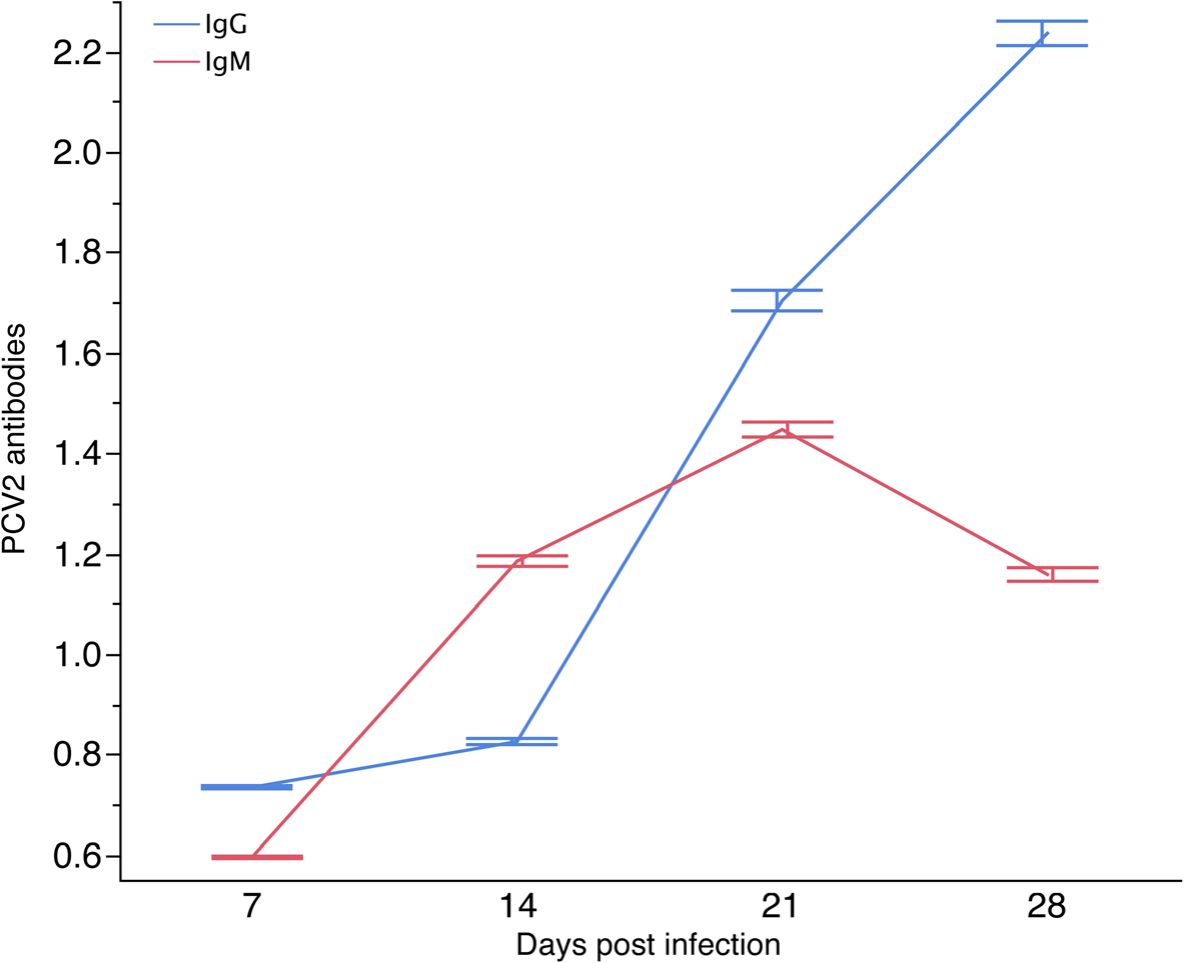
**Variation across time points in antibody response IgM and IgG following experimental challenge with PCV2b.**

**Figure 5:**
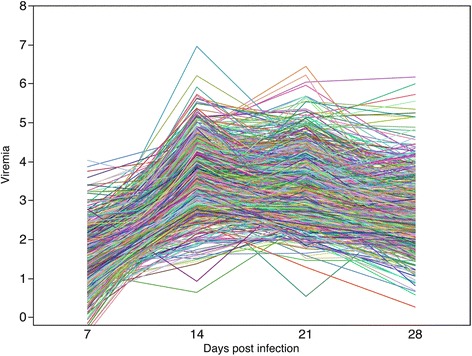
**Individual profiles for PCV2b viremia** * **following experimental challenge with PCV2b.** Individual profiles across time points demonstrate variation in magnitude and time of viral surge. *measured as number of PCV2 genome copy number/ml of serum (log10).

**Figure 6:**
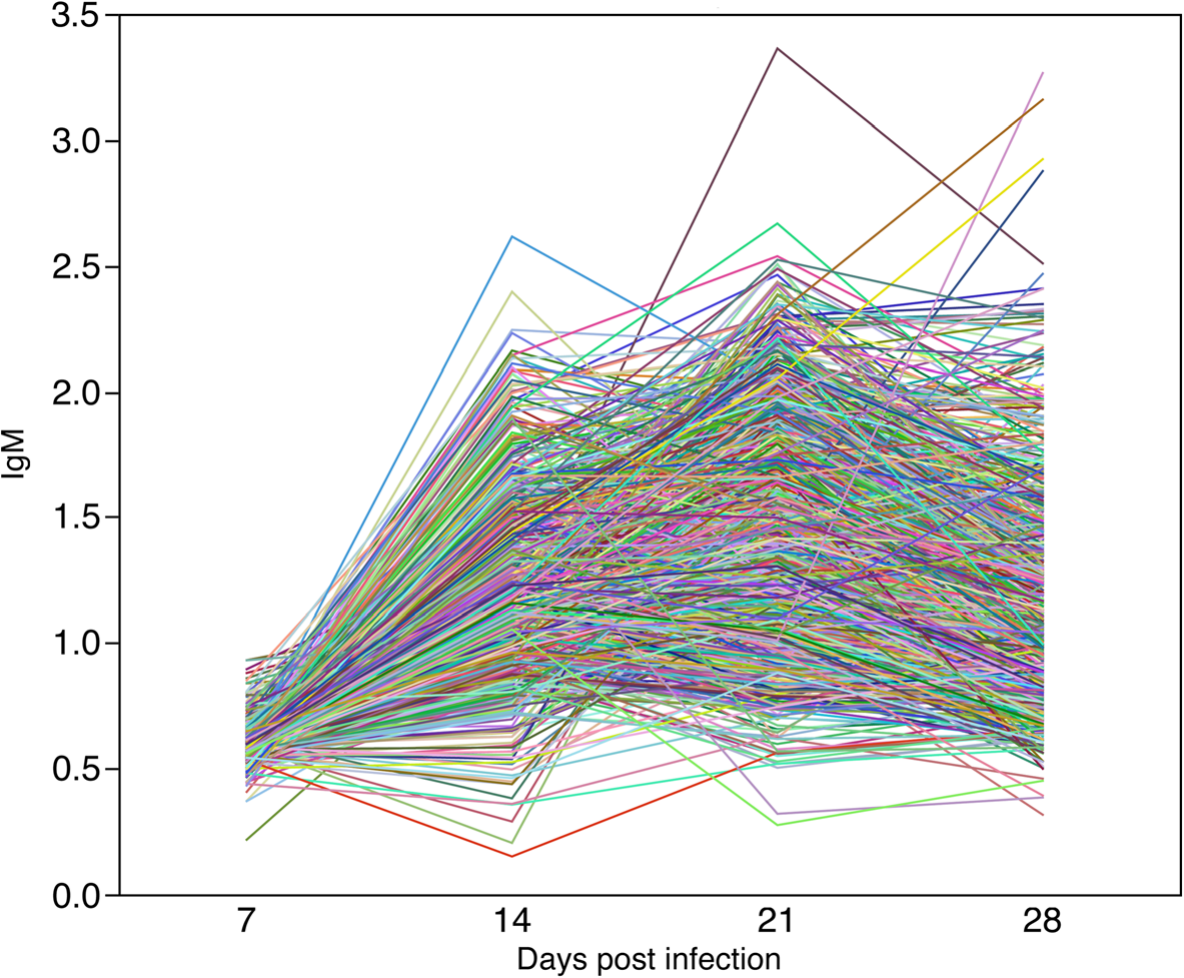
**Individual profiles for PCV2b-specific IgM antibody following experimental challenge with PCV2b.** Individual profiles across time points demonstrate variation in magnitude and time of immune response.

Viremia began to increase at d 7, and peaked at d 14, followed by a decline (Figure 3) with PCV2-specific antibodies following the same trend (Figure 4). Similar patterns were observed in other studies [5,19,20]. Individual viremia profiles across time points demonstrate that viral replication varies in the magnitude and time of viral surge (Figure 5); a similar trend was observed for IgM (Figure 6). As a result, viral load was also characterized by substantial variation across pigs (Table 2). The time-point with the largest variation in viremia was d 21. Individuals that reached early the maximum viremic value tended the have a lower overall viral load (P < 0.05). For example, the average viral load was lower in the pigs that reached the maximum viremia at d 14 (68.1) compared to those reaching the maximum viremia at d 21 (70.4)(P < 0.05).

**Figure 7:**
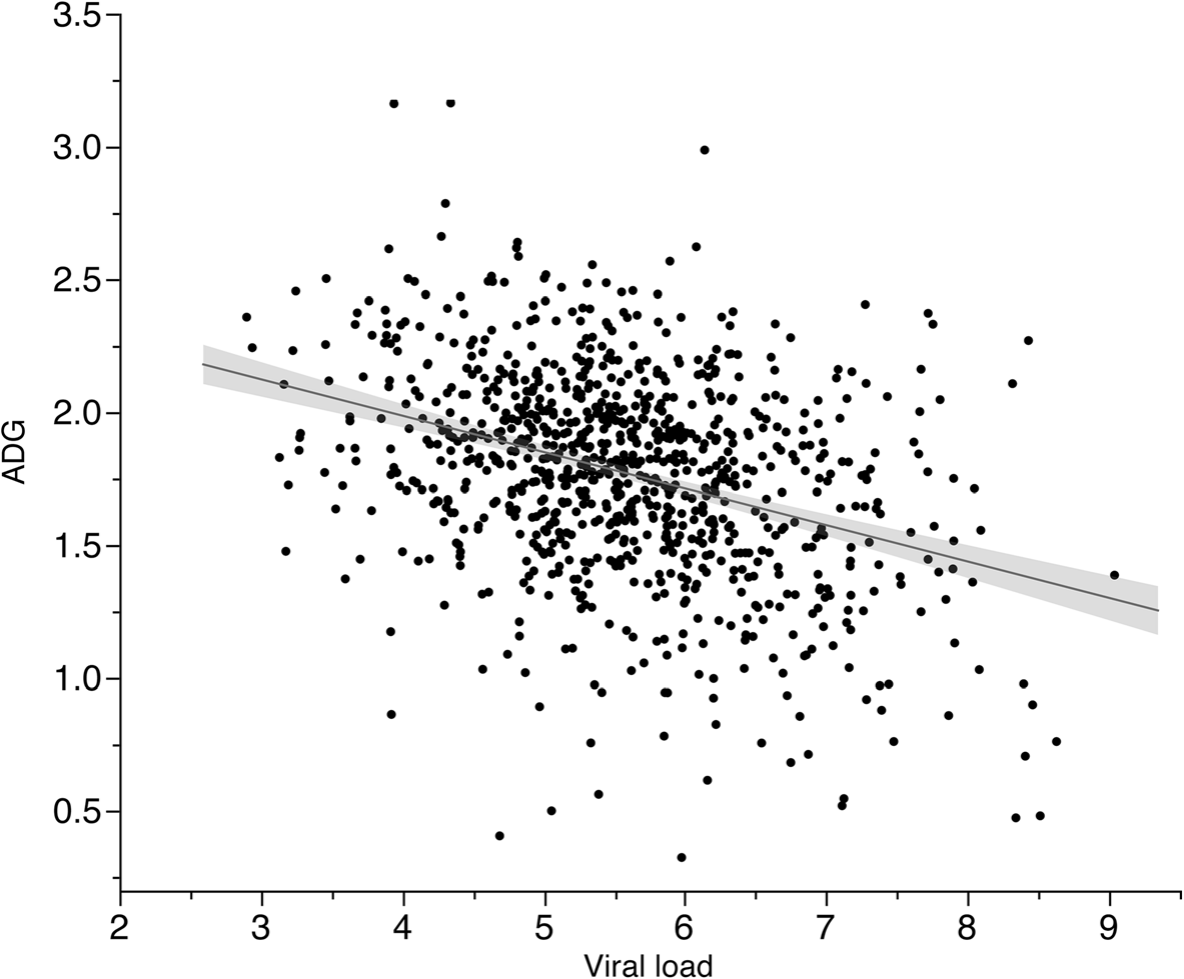
**A moderate negative correlation was detected between PCV2b viral load – X and overall ADG - Y (r = − 0.35,** ***P*** **< 0.0001) here represented as Z scores.** Each dot represents an individual pig. Upper left group included pigs that maintained low PCV2b viral load and fast growth and compared to the bottom right group which included pigs that expressed high viral load and slow growth during 28 d PCV2b challenge.

Pigs that expressed high ADG during the challenge were less viremic. A moderate negative relationship was detected between viral load and overall ADG (r = − 0.35, *P* < 0.0001)(Figure 7). The largest correlation between viral load and weekly ADG was detected for ADG on wk 4 (r = − 0.39, *P* < 0.001). Also, moderate negative phenotypic correlations were observed between weekly viremia measures at at d 14, 21, and 28 and ADG on wk 3 and wk 4, and overall ADG (r = − 0.30 to - 0.39; *P* < 0.0001). The largest correlation value between weekly measurements was obtained between viremia at d 21 and ADG on wk 4 (r = − 0.39, *P* < 0.0001, Additional file 2: Table S2). Decreased growth during challenge may be an effect of the animal’s inability to clear the virus as a result of a weak immune system, which increases the possibility of acquiring a secondary infection. A study conducted by Meerts et al. [21] indicated that levels of PCV2 viremia influenced susceptibility to PMWS in both natural and experimental PCV2 infections.

**Figure 8:**
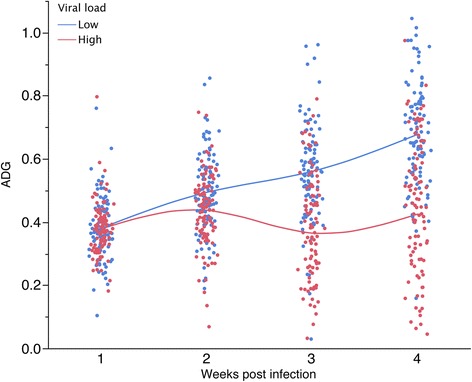
**Viral load influenced the decline in ADG following PCV2b infection.** The pigs with the lowest 10% level of viral load maintained a steady increased in weekly ADG (P < 0.0001) compared to the pigs that had the 10% largest viral load (P < 0.55). We predict that the growth of highly viremic pigs will be compromised even after the virus will be cleared.

The viral strain used for the experimental infections has a similar DNA sequence with a Canadian cluster of strains known to induce high mortality rates [12,13]. We expected that this pathogen would be responsible for declined growth during challenge and viremia would be an indicator of susceptibility. The ADG profile across time points was analyzed in pigs that expressed extreme phenotypes for viral load. In the first week of challenge, ADG was similar (P < 0.60) in pigs that displayed the lowest level of viral load (10%, n = 100, 0.38 g/d) compared to the pigs with the highest viral load (10%, n = 100, 0.39 g/d) (Figure 8). In the following weeks the low viremic group had a steady increased in weekly growth rate (P < 0.0001) with fold change in ADG of 1.30 (wk 2), 1.47 (wk 3) and 1.78 (wk 4) compared to wk 1. In contrast, the highly viremic pigs maintained a similar ADG (P < 0.55) during challenge having a fold change of 1.10 (wk 2), 0.94 (wk 3) and 1.09 (wk 4) compared to wk 1. Starting with wk 3 the individuals tend to form separate clusters based on their viral load and ADG (Figure 8).

The change in weekly ADG in the low viremic group was best described as a linear function (P < 0.05) as a result of a consistent increased in ADG during challenge. In contrast, the change in ADG in the highly viremic group was best described as cubic (P < 0.01), as a result of a lack of persistent pattern in ADG across time points. The difference in ADG between the two groups varied from 0.08 kg/d on wk 2 (P <0.005), 0.19 kg/d on wk 3 (P < 0.0001) and 0.25 kg/d on wk 4 (P < 0.0001). As a result, it is expected that in the highly viremic pigs the growth will be compromised even after the virus would be cleared. This indicates that viremia is the main driver of ADG decline following infection. In addition, the highly viremic group expressed higher IgM and IgG starting with d 14 and d 21 respectively, compared to low viremic group (P < 0.0001).

In a recent study we found small to moderate positive correlations between tumor necrosis factor – alpha (TNF-α) at d 21 and viral load (r = 0.25, P < 0.0001) and viremia (r = 0.23 to 0.34, P < 0.0001) during last weeks of challenge [22]. The TNF-α increased over time, reaching the peak at d 21. TNF-α is a pro-inflammatory cytokine involved in the activation of adaptive immunity to viral infections [23]. PCV2 infection reduces the capacity of plasmacytoid dendritic cells to induce interferon-α(INF-α) and TNF-α and as a result limits the maturation of associated myeloid dendritic cells [14,15] and subsequent immune response. At d 21 the highly viremic group showed higher level of TNF-α compared to low viremic group (P < 0.005).

Anecdotal evidence suggested that, in general, slow growth prior to the outcome of the disease challenge is associated with susceptibility. However, our previous research did not find significant relationships between ADG prior to infection with viremia during the first two weeks of challenge (r = 0.01 to 0.03, *P* > 0.63) or with viral load (r = −0.02, *P* = 0.73) [7].

### PCV2b-challenged pigs expressed variation in immune response

Previous reports indicated that most of the PCV2 infected pigs demonstrate subclinical symptoms in both natural and experimental infections since antibodies produced by the humoral immune response and other unexplained mechanisms are able to clear the virus [21]. The profile of PCV2 specific antibodies, IgM and IgG, were comparable with previous experimental infections [5,11] (Figure 4).

Similar to viremia, individual profiles of IgM across time points varies in the magnitude but also in the time of induction of the humoral response to PCV2b (Figure 6). The profile obtained for early responders precedes that of late responders by approximately one week. The IgM titres in early responders began to increase at d 7, and peaked at d 14 followed by a period of decline. In the late responders, IgM titres began to increase at d 14, and peaked at d 21 followed by slowly declining titers. Across all samples, the time point associated with the largest variation in IgM occurred at d 21. Individuals that reached early the highest IgM value tended to have a lower overall viral load (P < 0.009). For example, the average viral load was lower in the pigs that reached the highest IgM at d 14 (66.3) compared to those reaching the highest IgM at d 21 (69.7, P < 0.001) and d 28 (69.9, P < 0.05).

IgG titres started to increase in most pigs at 14 dpi, peaking at d 21 and d 28 (Figure 4). As expected, the time point associated with the largest variation in IgG occurred at d 28 (Table 2).

Pair-wise correlations between PCV2 specific antibodies and ADG were relatively low and varied between - 0.14 (IgM d 28 and ADG wk 4, *P* < 0.0001) and 0.08 (IgM d 14 and ADG wk 1, *P* < 0.05) for IgM and - 0.02 (IgG d 21 and ADG wk 4, *P* < 0.51) and 0.11 (IgG d 14 and ADG wk 3, *P* < 0.001) for IgG (Additional file 2: Table S2). Fort et al. [24] showed that IgG was primarily associated with neutralizing capacity (r = 0.85, *P* < 0.001) while IgM had limited effect on PCVAD progression. Decreased levels of neutralizing antibodies resulted in an increase in PCV2 viral replication and progression of PMWS [25].

As expected, positive moderate relationships were observed between viremia and antibody response; significant small to moderate correlations were determined between viral load and IgG starting with d 21 (r = 0.26 to 0.27, *P* < 0.0001) and IgM starting with d 14 (r = 0.32, *P* < 0.0001) (Additional file 2: Table S2). While in the current study the pigs were exposed only to PCV2b, we hypothesize that variation in immune response could be the source of reduced PCVAD susceptibility in some pigs. Specifically, certain host genotypes may have the ability to inhibit the role of PCV2 as an immunomodulator, reducing susceptibility to coinfection.

## Conclusions

There are numerous challenges in developing management programs to prevent PCV2 infections. PCV2 is exceptionally stable and disinfection is difficult. In addition, PCV2 is prevalent worldwide, and nearly every swine operation in the United States has already been exposed to the virus. The fact that PCV2 is extremely stable and is present globally creates opportunities for viral transmission, making it challenging to produce specific-pathogen-free animals. The use of vaccines for PCV2 has proven successful but increases the cost of production. Moderate negative relationships were determined between viremia and ADG while the relationship between PCV2 specific antibodies expressed by experimental animals and ADG were limited. While some of these relationships were reported by previous studies, some of those experiments were limited in size and focused mainly on pure breeds. In contrast, this data set has a size that provides sufficient power to estimate accurate relationships between indicators of PCVAD susceptibility. In addition, most of the genetics used in this research represent the maternal side of production herds, populations that are subjected to a more pathogen-challenging environment compared to nucleus herds. Interestingly, this study uncovered substantial variation in the time of initiation and magnitude of viremia across a diverse range of the genetic resources used in North America. Dissection of the underlying molecular events responsible for the observed differences in viral replication and immune response could provide a better understanding of the swine immune system and the host factors responsible for PCVAD susceptibility. The mechanism of how certain pigs inhibit viral replication is not well understood, and further research is needed to gain a better understanding of the role of host genetics in response to infection.

## Methods

### Experimental design: animals, diets, and housing

The experimental procedures used during this study were reviewed and approved by the Institutional Animal Care and Use Committee of the University of Nebraska-Lincoln (UNL). The pigs used for the experimental challenge (n = 974) were distributed over time in nine batches (B1 to B9) ranging from 81 to 141 pigs per batch. Barrows were used in all of the batches except B2 and B3, which consisted of both barrows and gilts. The pigs originated from the UNL Swine Research Unit, and members of the PigGen Canada Consortium, being generated by seven different genetic programs. Pigs were derived from 320 litters with an average representation of 3.04 pigs per litter. The UNL Swine Research Unit provided maternal and terminal crossbred pigs (B1 to B4; n = 386) based on Large White, Landrace, and Duroc from a commercial source as well as the Nebraska Index Line (Additional file 1: Table S1), which has been selected since 1981 mainly for traits related to increased litter size. Five members of PigGen Canada consortium (www.piggencanada.org) provided maternal crossbred pigs (B5 to B9, n = 588) based on Large White and Landrace genetics.

All experimental pigs were colostrum fed and raised under similar conditions.

Dams of the experimental pigs were vaccinated for PCVAD at 2–3 weeks of age. In addition, the source farms have vaccination programs for Porcine parvovirus (PPV), Erysipelothrix rhusiopathiae, Clostridium perfringens type C, Leptospirosis, Colibacillosis and regular testing for Porcine reproductive and respiratory syndrome virus (PRRSV). Before experimental infection, each pig was evaluated and tested negative for the presence of PCV2, based on PCV2–specific antibodies and PCV2 viremia [7]. Specifically, each pig was tested for levels of IgG and IgM PCV2 specific antibodies using ELISA. Pigs used for the experimental infection had to show a sample/positive ratio (S/P) lower than 0.3 for passive (maternal) IgG antibodies and lower than 0.4 for IgM. High levels of passive antibodies were associated previously with reduced susceptibility to PCVAD [17]. The pigs were also tested for the presence of viral DNA using quantitative real-time PCR (qPCR) as described [7]. Pigs that demonstrated negligible S/P levels and tested negative for PCV2 viral DNA were transferred to the UNL Animal Science Complex for the experimental challenge.

Throughout the experimental infection, pigs were housed in one experimental room containing identical pens. Pigs were randomly assigned to a pen, which consisted of both slatted and solid surface flooring. Each pen provided approximately 0.65 square meters of floor space per pig. All pigs were allowed ad libitum access to a standard balanced diet that met or exceeded nutritional requirements for the respective age. Upon arrival at UNL facilities, each pig received a preventative dose of 0.8 ml of Baytril 100 (Bayer Animal Health) to treat for potential bacterial pathogens.

### PCV2b isolate and experimental infection

The PCV2b strain (UNL2014001) used for the experimental infection was isolated from a pig with Post-weaning Multisystemic Wasting Syndrome (PMWS) symptoms, a PCVAD syndrome, and cultured in swine testicular lines as described [7]. Experimental pigs were inoculated with a titer of 10^4.0^ TCID_50_/ml of the PCV2b strain both intranasally and intramuscularly. The average age of infection across all batches was 43 days of age. Daily observations were conducted during the 28 days experimental period to monitor and detect clinical symptoms. In addition, weight and blood samples were collected a 0, 7, 14, 21, and 28 days post infection (dpi).

### Serology: PCV2-specific antibodies and viral DNA quantification

Blood samples were collected in sterile Monoject red stopper blood collection tubes containing no additives (Tiger Medical) and separated into BD Vacutainer tubes (Becton Dickinson) and Tempus blood RNA collection tubes (Life Technologies). The serum was obtained by centrifugation at 2,350 *g* for 15 minutes at 4°C.

Enzyme-linked immunosorbent assay (ELISA; Ingenasa) was used to evaluate the levels of PCV2 specific antibodies, IgG and IgM, in serum. The concentration of PCV2 specific antibodies were normalized based on positive control values corrected by 0.3 fold for IgG and 0.4 fold for IgG. An IgG or IgM normalized value greater than one differentiated PCV2 positive from negative pigs according to the manufacturer of the ELISA kit. Protein serum level of tumor necrosis factor – alpha (TNF-α) was quantified using ELISA assays (R&D Systems, Inc.).

Viremia or estimates of viral copy counts in blood were measured for each pig and time point as described [7]. Viral DNA was first isolated from serum samples using QIAamp DNA Minikit (Qiagen) and was then quantified by qPCR using TaqMan Master Mix and ABI 7900 Real Time PCR System (Life Technologies). Area under the curve (AUC) was used to evaluate the total viral load for each pig throughout the entire experimental challenge period based on an algorithm that uses viremia levels determined at each time point (0, 7, 14, 21, and 28 dpi) to fit a smooth curve over the 28 d infection period and summed the areas in increments of 0.01 time units [26].

### PCV2b Sequencing

PCV2b viral genomic DNA was isolated using QIAamp DNA Minikit and amplified using GoTaq Flexi DNA Polymerase (Promega). The PCR products were purified using ExoSAP-IT (USB Corporation) and sequenced using dye terminators and ABI PRISM 3100 Genetic Analyzer (Life Technologies). The assembled sequence (GenBank accession number: KP016747) was aligned to the publicly available PCV2 genome sequences using CLUSTALW2 [27].

Following the experimental challenge, viral genomic DNA was isolated from random, high and low viral load pigs (n = 18) representing most of the batches and sequenced to validate the genetics of PCV2b strain used for experimental infection.

### SLAII haplotyping

The Swine leukocyte antigen (*SLAII*) haplotypes were determined by sequencing the coding area of the *DQB1* gene, a member of SLAII gene complex, and compare the obtained sequences to the reference haplotypes. Specifically, the coding area of *DQB1* was amplified in 27 pigs representing all batches using GoTaq Flexi DNA Polymerase. The PCR products were purified using ExoSAP-IT and sequenced using dye terminators and ABI PRISM 3100 Genetic Analyzer. Individual sequences from each pig were compared to reference haplotypes sequences obtained from Immuno Polymorphism Database (www.ebi.ac.uk/ipd/mhc) and *DQB*1 *SLAII*-specific haplotypes were assigned for each pig.

### Statistical analysis

The means and measures of variability were estimated for each trait across time points. The pair-wise correlation between traits was performed using adjusted phenotypes. The correction of the phenotypes was based on residuals estimated from a linear mixed model treating batch as a fixed effect, litter and pen as random effects, age at infection and passive IgG as covariates. Since the traits profiled were clearly related to each other, the multiple testing was handled by considering the correlations significant if the P < 0.0001. The influence of dam parity on the level of passive IgG in experimental piglets prior to infection was determined based on a linear mixed model with batch and parity as fixed effects, litter as random effect and age at infection as a covariate.

## Additional files

Additional file 1: Table S1. Genetics of the resource population used in the experimental infection with PCV2b. Additional file 2: Table S2. Pair-wise correlations between the adjusted phenotypes of the indicator traits of PCVAD susceptibility following an experimental infection with PCV2b.
